# Pathogenicity and virulence of *Bordetella pertussis* and its adaptation to its strictly human host

**DOI:** 10.1080/21505594.2021.1980987

**Published:** 2021-09-30

**Authors:** Thomas Belcher, Violaine Dubois, Alex Rivera-Millot, Camille Locht, Françoise Jacob-Dubuisson

**Affiliations:** Univ. Lille, CNRS, Inserm, CHU Lille, Institut Pasteur de Lille, U1019 - UMR 8204 - CIIL - Center for Infection and Immunity of Lille, Lille, France

**Keywords:** Pertussis, *Bordetella*, metabolism, virulence factors, evolution, adaptive immunity, innate immunity

## Abstract

The highly contagious whooping cough agent *Bordetella pertussis* has evolved as a human-restricted pathogen from a progenitor which also gave rise to *Bordetella parapertussis* and *Bordetella bronchiseptica*. While the latter colonizes a broad range of mammals and is able to survive in the environment, *B. pertussis* has lost its ability to survive outside its host through massive genome decay. Instead, it has become a highly successful human pathogen by the acquisition of tightly regulated virulence factors and evolutionary adaptation of its metabolism to its particular niche. By the deployment of an arsenal of highly sophisticated virulence factors it overcomes many of the innate immune defenses. It also interferes with vaccine-induced adaptive immunity by various mechanisms. Here, we review data from *in* *vitro*, human and animal models to illustrate the mechanisms of adaptation to the human respiratory tract and provide evidence of ongoing evolutionary adaptation as a highly successful human pathogen.

## Graphical Abstract

**Figure:**
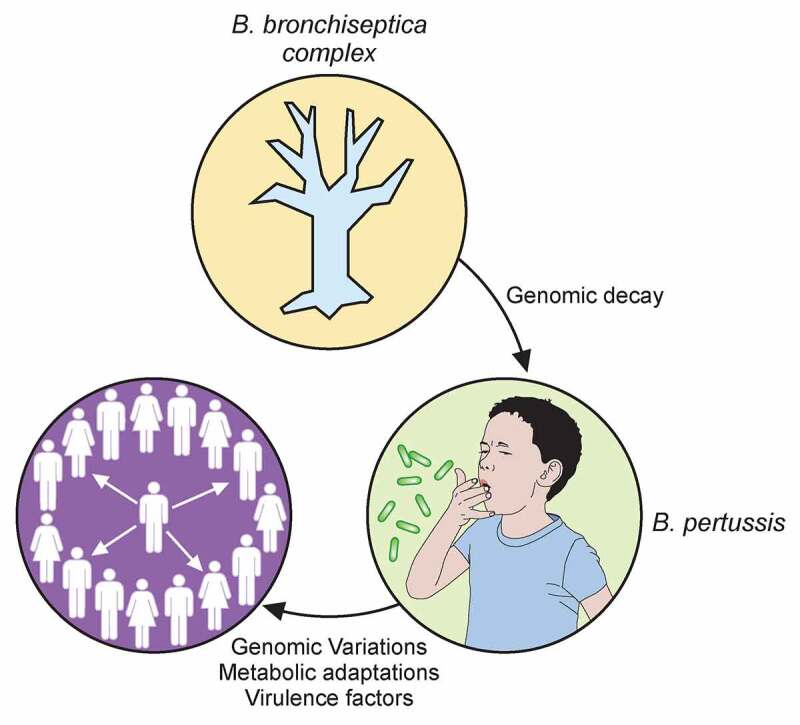

## Introduction

*Bordetella pertussis* is the main causative agent of pertussis or whooping cough, an acute respiratory disease that severely affects young children [1], but also constitutes a significant disease burden in adolescents and adults. Despite a very high global vaccination coverage close to 85% [2], the most recent models put estimates at 24 million cases annually causing more than 160,000 deaths in children less than 5 years of age. Nevertheless, many cases remain undiagnosed. This disease overwhelmingly affects low-income countries. However, in spite of strong immunization policies since the 1950s, even industrialized countries have failed to eradicate the disease. In fact, over the past 10 years, a reemergence of this pathogen has been reported in several countries, including more than 15,000 cases diagnosed in 2019 in the United States. For children that become infected under 6 months of age, the hospitalization rate is over 40%. Several factors might explain the upward trend of pertussis, including improvements of diagnostic techniques [3], vaccine-driven antigenic drift of circulating strains [4], the short duration of protection induced by acellular pertussis vaccines (aPV) [5], and the failure of the current vaccines to prevent *B. pertussis* infection and transmission [6].

Whooping cough is highly contagious and transmitted by aerosol droplets [7]. In the pre-vaccination era, the reproduction number R0 was estimated at 12 to 17, similar to that of measles (R0 = 12–18 in unvaccinated populations), but much larger than that of smallpox (R0 3.5–6) [8–10]. This makes *B. pertussis* one of the most contagious pathogens. Furthermore, whereas the R0 of measles dropped to almost zero in vaccinated populations, that of pertussis remained between 5 and 6 in areas with high vaccination rates, such as in Europe [11], implying that *B. pertussis* persists and circulates even in populations with high vaccination coverage.

### The evolutionary history of B. pertussis

The genus *Bordetella* contains many species, most of which have been isolated from severely immunocompromised individuals. Only a few of them, including *Bordetella petrii*, have so far only been isolated from natural milieus [12–14]. This contrasts with the closely related genus *Achromobacter*, which comprises mostly environmental species and only a few opportunistic pathogens. Among the 16 known *Bordetella* species, 3 closely related species that form the “*Bordetella bronchiseptica* complex”, *B. bronchiseptica, B. pertussis* and *Bordetella parapertussis* have attracted most of the attention, as they are bona fide pathogens for humans and/or other mammals.

Given the paucity of information on most environmental *Bordetella*, studies on the evolutionary trajectory in this genus have mainly focused on the *B. bronchiseptica* complex, even though they are not the only professional pathogens of the genus [15]. In the *B. bronchiseptica* complex, the two-component regulatory system BvgAS enables the bacteria to switch from a virulent Bvg^+^ phase in which virulence-associated genes (*vag*s) are expressed, to an avirulent Bvg^−^ phase, in which they are silenced [16]. The avirulent phase is also characterized by the expression of a set of virulence-repressed genes (*vrg*s), including those coding for a flagellum and for metabolic pathways allowing growth in nutrient-limited media [15,17,18].

With its complex life cycle alternating between the soil and mammalian hosts, *B. bronchiseptica* is a good model to understand the transition from one environment to the other and how Bvg regulation is instrumental to this adaptation [15]. The finding that *B. bronchiseptica* survives and multiplies within amoebae, such as *Dictyostelium discoideum*, was key to understanding the selective pressure that has led to the acquisition of its virulence regulon [19]. Amoebae are protozoa that predate on bacteria as a source of nutrients, and their digestion mechanisms are very similar to the bactericidal mechanisms of mammalian phagocytic cells [20].

Similar evolutionary trajectories have been described for other pathogens, including *Pseudomonas, Legionella, Listeria, Francisella, Burkholderia, Mycobacteria* and others [21–25]. With their reduced genomes compared to that of the broad host range pathogen *B. bronchiseptica*, human-restricted *B. pertussis* and *B. parapertussis* are no longer able to survive in the environment [26]. *B. pertussis* also lost the ability to survive within amoeba and is considered primarily an extracellular pathogen, although some reports have described its admittedly limited capacity to survive within respiratory epithelial and phagocytic cells, which may be an evolutionary remnant [27–29]. Instead, *B. pertussis* and *B. parapertussis* have adopted specific infection strategies adapted to their cognate hosts.

*B. bronchiseptica* infects various mammals, and serological investigations of domestic animals have indicated that it is an almost ubiquitous pathogen [30–32]. Paradoxically, humans are rarely infected with this bacterium [33], whereas they are readily colonized by *B. pertussis* and *B. parapertussis* [34]. Unlike *B. bronchiseptica*, which is not very virulent or contagious in animal populations but instead causes chronic infections, *B. pertussis* and, to a lesser extent *B. parapertussis*, rely on high levels of virulence and transmissibility [35]. They cause acute, relatively short-lived infections, except in individuals unable to control the infection. It is likely that *B. pertussis* and *B. parapertussis* emerged and adopted this life style thanks to the evolution of the social organization of humans [35,36], whose population density has steadily increased throughout history. Host abundance and proximity enable easily-transmitted bacteria to persist in the population. Conversely, lower-density populations select pathogens able to persist in their host for longer periods, as they cannot quickly colonize new hosts.

It has been proposed that *B. pertussis* and *B. parapertussis* emerged independently from a common ancestor similar to current-day *B. bronchiseptica* and adopted similar modes of infection of their human host [37]. While a set of adaptive mutations enabled the former two species to cause acute infections in humans, the wide range of host species available to *B. bronchiseptica* limited the evolutionary pressure on the latter to adapt to human hosts. Its poor ability to infect humans may be due to the principle that two populations cannot survive within the same ecological niche if their requirements are similar [38]. Therefore, indirect rather than direct competition between bacteria may be mediated by the host. Since the three species are genetically extremely close, infection with any of them may trigger the development of adaptive immune defenses targeting virulence factors common to all three. In this context, *B. pertussis* and *B. parapertussis* appear to be favored by their infection strategy, as acute disease and rapid spread allow these bacteria to persist in the population. Since a large part of the human population has developed immune defenses against the virulence factors of the *B. bronchiseptica* complex, *B. bronchiseptica* is disadvantaged by its lower level of transmission.

The initial emergence of *B. pertussis* and the disappearance of *B. bronchiseptica* in humans are thought to have been favored by modifications of the immunogenic profile of *B. pertussis* to partially circumvent preexisting immunity against *B. bronchiseptica*. As such, *B. pertussis* has replaced lipopolysaccharide (LPS) with lipooligosaccharide (LOS) and lost its flagellum, two highly immunogenic components (see below). Furthermore, *B. pertussis* produces large amounts of pertussis toxin (PTX) to limit the recruitment and the activation of phagocytes, as it lost most genes required to survive phagocytosis. Thereby, it has taken advantage of the limited anti-PTX immune response elicited by *B. bronchiseptica* infection, whose lifestyle appears to require at most very low levels of PTX. These differences may represent evolutionary steps that have allowed *B. pertussis* to partially escape the immunity induced by the competing species

## An arsenal of virulence factors

### Regulatory network in B. pertussis

**Figure 1.**
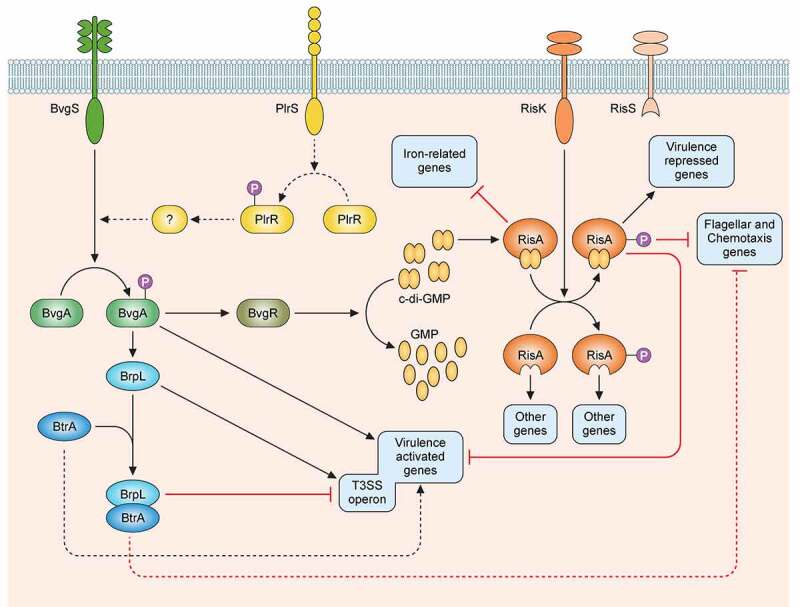
**Regulatory network of the virulence factors in *B. pertussis***. BvgS is a sensor-kinase that is active by default at a temperature of 37°C. Its autophosphorylation is followed by the phosphorylation of its cognate response regulator BvgA. Phosphorylated BvgA triggers the expression of vir-activated genes (*vags*). BvgS responds to chemical stimuli such as sulfate or nicotinate ions by switching off the phosphorylation cascade, which increases the expression of vir-repressed genes (*vrgs*). BvgR is the product of a *vag* and hydrolyzes c-di-GMP to GMP. c-di-GMP affects the activity of the RisAK two-component system by binding to the response regulator RisA. The sensor-kinase RisS is truncated and nonfunctional in *B. pertussis*, and the partner of RisA is the sensor-kinase RisK. The targets of RisA depend on its phosphorylation and on the concentration of c-di-GMP. Non-phosphorylated RisA bound to c-di-GMP represses the expression of iron-related genes. With both c-di-GMP and phosphorylation RisA induces the expression of the *vrgs* and represses that of the *vags* and of the mobility genes, whereas in the absence of c-di-GMP, RisA induces the expression of other sets of genes depending on its phosphorylation. The sensor-kinase PlrS responds to CO_2_ by phosphorylating the response regulator PlrR. The regulon of PlrSR is unknown, but one of its target(s) interact(s) directly or indirectly with the BvgAS system in an uncharacterized manner. BrpL is a *vag* coding for a sigma factor that triggers the expression of the T3SS. BtrA antagonizes the activity of BrpL by titrating it in a BtrA/BrpL complex, which results in the repression of the T3SS and the mobility genes and the overexpression of certain *vags*. The dotted arrows represent interactions identified in *B. bronchiseptica* and suspected in *B. pertussis.*

Evolution has endowed *B. pertussis* with many virulence factors that make it an extremely contagious pathogen. The virulence regulon of *B. pertussis* contains more than 200 genes, which are controlled in a coordinated manner by the two-component system BvgAS [18,39,40] (Figure 1). In the Bvg^+^ phase the system is active by default. The sensor-kinase BvgS requires no activating ligand to induce its auto-phosphorylation and the subsequent transfer of the phosphate group to the transcriptional activator BvgA. Phosphorylated BvgA binds to the promoter regions of the *vag*s and transactivates their expression. These genes notably encode toxins and adhesins and their secretion machineries. They also include *bvgR*, encoding a c-di-GMP phosphodiesterase, which indirectly down-regulates the expression of the *vrgs*. In laboratory conditions, BvgS responds to chemical stimuli, such as nicotinate and sulfate ions, by turning off its auto-phosphorylation, which shifts the bacteria to the Bvg^−^ phase [41]. Low temperatures (<25°C) also shift *B. bronchiseptica* to the Bvg^−^ phase, which involves remodeling of membrane lipid composition, whereas *B. pertussis* responds only partially to low temperatures [42]. An intermediate Bvg^i^ phase also exists at intermediate concentrations of modulators, and possibly at intermediate temperatures corresponding to those of the nasal cavity. In the Bvg^i^ phase, certain adhesins are produced, but not the toxins [43,44]. The signals to which BvgS might respond *in* *vivo* are not known [45].

Recent studies have revealed a more complex picture of virulence regulation in *B. pertussis*, involving several additional systems [46] (Figure 1). The two-component RisA/S system is an ortholog of EnvZ-OmpR, known to regulate responses to osmolarity, motility and in some cases virulence in many Gram-negative bacteria [47]. *B. pertussis* has a truncated nonfunctional RisS sensor-kinase, but another sensor-kinase, RisK, phosphorylates the response regulator RisA [48]. Phospho-RisA triggers the differential expression of many genes, including the *vrg*s, depending on the intracellular concentration of c-di-GMP, which is hydrolyzed by BvgR when the bacterium is in the Bvg^+^ phase [49].

An additional two-component system, PlrRS, is thought to be involved in the colonization of the lower respiratory tract [50,51]. This system reacts to the concentration of CO_2_ and modulates the expression of certain *vag*s, which might be useful in minimizing the inflammatory response of the host [46].

In addition to those two-component systems, sigma/anti-sigma factors regulate specific aspects of virulence. One such system is the BtrA/BrpL pair involved in the regulation of the type III secretion system (T3SS) [52]. However, it also intersects with the regulation of several other virulence factors, such as filamentous hemagglutinin (FHA), adenylate cyclase toxin (ACT) and pertactin (Prn). BtrA/BrpL is sensitive to *in* *vitro* stresses, such as glutamate limitation and the oxidation state of the culture medium [53,54]. The BrpL sigma factor is one of the *vag*s.

### Major virulence factors of B. pertussis

Many of the *B. pertussis* virulence factors have immunomodulatory properties that play important roles in host-pathogen interactions, including adhesins and toxins (Table 1). Several virulence factors, such as FHA, PTX, Prn and fimbriae, are included in the current aPV.

**Table 1.** Major virulence factors of *B. pertussis.*

Major virulence factors				
Regulators	Type	Stimuli	Regulon	
BvgAS	TCS*	In lab: nicotinate, MgSO_4_	[40,48]	
RisAK	TCS	Unknown	[49,107]	
BtrA	Sigma factor	In lab: oxidative conditions, iron starvation, glutamate limitation …	[52]	
PlrRS**	TCS	CO_2_	Unknown [51]	
RseA/RpoE	Sigma factor	Envelope stress	[55]	
Adhesins	Name	Structure	Secretion	
FHA	Filamentous hemagglutinin	β helix [56]	Two-Partner Secretion [67]	
Prn	Pertactin	β helix [57]	Autotransporter [72]	
Fim	Fimbriae	Polymer of pilin subunits [58]	Chaperone-usher pathway [59]	
Toxins	Name	Structure	Secretion mechanism	Activity
PTX	Pertussis toxin	AB_5_-type exotoxin [78]	Type IV secretion system Ptl [79]	ADP ribosylation of G proteins [80]
ACT	Adenylate cyclase	RTX toxin [83]	Type I secretion system CyaBDE [83]	Pore forming and calmodulin-dependent adenylate cyclase [83]
TCT	Tracheal cytotoxin	Disaccharide-tetrapeptide [60]		Peptidoglycan fragment [85,86] triggering an inflammatory response
DNT	Dermonecrotic toxin	Homologous to *Pasteurella multocida* toxin [61]	Not secreted [62]	Transglutaminase activating Rho GTPases [63]
BteA/BopC	T3SS effector	[195]	Type III secretion system [64]	Cytotoxicity, unknown mechanism
BopN	T3SS effector	Homologous to *Yersinia* effector [65]	Type III secretion system [64]	Cytotoxicity, unknown mechanism

* Abbreviations: TCS, two-component systems; RTX, repeats in toxin; T3SS, type three secretion system

Numbers in brackets refer to references.

FHA is a major adhesin of the genus *Bordetella* [66]. It forms a long beta helix and results from the proteolytic maturation of the 370-kDa precursor FhaB. It is secreted by a two-partner secretion system (TPS) [67] and mediates adherence to ciliated cells in the respiratory tract [68]. FHA also mediates bacterial aggregation [69].

The *Bordetella* fimbriae are type I pili. Two fimbrial serotypes are produced by *B. pertussis*, encoded by the *fim2* and *fim3* genes, respectively [70]. The pili are assembled via a chaperone-usher pathway, with the FimD subunit found at the distal end of the pili made up of Fim2 or Fim3 protein monomers. The fimbriae are involved in the adherence to the host’s ciliated cells [71].

Prn is an auto-transporter [72]. Its biological role is poorly understood. Some *in* *vitro* studies have provided evidence that it mediates adherence to ciliated cells of the epithelium [73], but its deletion does not affect mouse lung colonization by *B. pertussis* [74], and many recent clinical isolates no longer produce it, most likely because of vaccine-induced selective immune pressure [4] (see below). Additional autotransporter proteins found in *B. pertussis* include adhesins, such as the tracheal colonization factor Tcf [75] and factors involved in resistance to complement, such as BrkA [76] and Vag8 [77].

PTX is an AB_5_-type holotoxin of 105 kDa [78] secreted by the Ptl type IV secretion system [79]. The A subunit expresses an ADP-ribosyltransferase activity, and the B moiety is a pentamer responsible for binding of the toxin to the target cells [80]. The targets of the ADP-ribosyltransferase activity are the α subunits of many Giα and Goα subunit isoforms of trimeric G proteins, leading to dysregulation of several signaling pathways [81]. Notably, ADP-ribosylation of the G protein that transduces the adrenergic β-receptor-triggered negative regulation of the membrane-bound adenylate cyclase reverses this inhibition, resulting in unregulated cAMP production [82].

ACT is a member of the repeats in toxin (RTX) family. It is secreted via a Type I secretion system. The C-terminal part of ACT contains the RTX domain as well as 4 hydrophobic segments, which mediate attachment to target cells and form a pore in the plasma membrane [83,84]. Its N-terminal domain expresses a calmodulin-dependent adenylate cyclase activity, which converts ATP into cAMP. This toxin therefore causes ionic imbalances in the target cells through pore formation, as well as deregulation of their signaling pathways due to accumulation of cyclic AMP and depletion of ATP [83,84]. ACT specifically targets complement receptor (CR)3-expressing sentinel cells of the myeloid lineage [83].

Another major *B. pertussis* toxin is tracheal cytotoxin (TCT), a disaccharide-tetrapeptide produced during the remodeling of the bacterial peptidoglycan. Most Gram-negative bacteria recycle this fragment, but *B. pertussis* does not re-import it into the cytoplasm and, instead, releases it in large quantities to induce a pro-inflammatory response [85] and overexpression of the inducible nitric oxide synthase [86], triggering the destruction of ciliated cells in the upper respiratory tract [87].

Dermonecrotic toxin permanently activates the Rho GTPase of target cells by polyamination and deamidation of glutamines [88,89]. However, its biological role in pertussis pathogenesis is poorly understood because it is not secreted but remains within the bacterial cytoplasm. Currently, no study has been able to demonstrate how this toxin is delivered to target cells. However, recently, its cellular receptor was identified as the T-type voltage-gated Ca^2+^ channel CaV3.1, a protein particularly present in the nervous system [90].

Bordetellae also possess a BvgAS-regulated T3SS. The effector protein BteA (also known as BopC) was shown to cause necrosis of certain cell types *in* *vitro* [91]. A more recent study carried out with *B. bronchiseptica* has demonstrated the action of BteA on signaling mechanisms of the actin cytoskeleton, inducing necrosis of certain cell types but also the inhibition of phagocytosis by macrophages [92]. In *B. pertussis*, a specific amino acid substitution in the BteA protein [93] appears to decrease its cytotoxicity. BopN, another effector, was reported to be necessary for the cytotoxicity of BteA in certain epithelial cell lines [94]. BopN harbors nuclear targeting signals, suggesting its involvement in gene regulation of target cells [95].

## Metabolic adaptation and pathogenicity

### The basic metabolism of B. pertussis

The metabolism of *B. pertussis* has been mostly studied *in* *vitro*, essentially due to the need to identify optimal *B. pertussis* growth requirements for vaccine production. *B. pertussis* grows slowly, even under optimal conditions. It is not able to utilize sugars as carbon source [96], as three genes encoding enzymes involved in the glycolytic pathway (glucokinase, phosphofructokinase and fructose-1,6-bisphosphate) are lacking in its genome [97]. The molecules most readily metabolized by *B. pertussis* are amino acids, the most efficient of which is glutamate [96]. *B. pertussis* can be grown with glutamate as the sole carbon source and cysteine as a source of sulfur, demonstrating that all building blocks of the cell can be synthesized from just a few sources [98]. Glutamate enters the TCA cycle via glutamate dehydrogenase, from which it can feed into different branches, such as gluconeogenesis for synthesizing sugars of LOS and the capsule, or anabolism of other amino acids. Although it was initially assumed that *B. pertussis* had an incomplete TCA cycle, since it could not grow on citrate, all genes required for a fully functional TCA cycle are present in its genome [97] and are expressed. Furthermore, their products are functionally active [99].

Compared to *B. pertussis, B. bronchiseptica* is able to utilize a wider range of substrates, including sugars, which is compatible with its environmental niche [97]. This difference in metabolism between the two species may also be related to the difference in host specificity. Since *B. pertussis* is highly host restricted, it likely has adapted its metabolism to take advantage of what is readily available in its specific niche of the human nasopharynx and may have lost the ability to metabolize various substrates, including sugars. Instead, it has retained a broad metabolic potential and is able to synthesize most of what is necessary for growth, with few exceptions, such as nicotinic acid [100] and glutathione.

Early difficulties in growing *B. pertussis* in the laboratory were due to inhibitory molecules present either in media or produced during growth, in particular fatty acids. Therefore, substances such as starch, blood or heptakis ((2,6-*O*-dimethyl) β-cyclodextrin) [101], are required in growth media to prevent growth inhibition by the presence of fatty acids [102,103]. Palmitic acids and other fatty acids were found in cell extracts of *B. pertussis* in concentrations likely to be inhibitory, suggesting that they are byproducts of growth. Interestingly, sensitivity to fatty acids appears to have been specifically selected for in *B. pertussis* and is not shared by *B. bronchiseptica*. The lack of *B. pertussis* growth in the presence of fatty acids can be overcome by restoring the functionality of an operon coding for an efflux pump whose gene is inactivated in the *B. pertussis* genome due to a frame-shift insertion [104]. The production of autoinhibitory free fatty acids during growth seems counterintuitive. It may be that growth within the infected host does not result in the production of these fatty acids in enough quantity to affect growth, or that fatty acids are washed away or neutralized during *in vivo* growth. This is suggested by the high bacterial densities (up to 10^8^ CFU/ml) that can be recovered from nasal washes of infected baboons [105], which do not include bacteria attached to the tissue and forming biofilms. Thus, the bacteria are not inhibited from reaching high densities *in vivo*. The *in vivo* relevance of the production of and sensitivity to fatty acids is not known and has not been explored.

### Metabolism with host perspective: Linking metabolism and virulence

The link between metabolism and *B. pertussis* virulence is not yet fully understood. However, it has been shown that *B. pertussis* virulence may depend on the medium used to grow the bacteria, since proteins are differentially secreted depending on the growth medium [106]. Whereas the most abundant proteins secreted in glutamate-rich as well as in glutamate-poor media are well-known virulence factors, a number of less abundant proteins, including some thought to be virulence associated were found uniquely to be secreted in glutamate-poor media [53]. Other proteins were found to be secreted only in glutamate-rich medium. Glutamate-sensitive protein secretion might reflect metabolic adaptation to physiological concentrations of glutamate during infection.

In addition to bona fide virulence genes, both *B. bronchiseptica* and *B. pertussis* regulate many metabolic genes via the BvgAS two-component system. In *B. bronchiseptica*, which can switch between mammalian hosts and environmental niches, functional *vrg*s are much more abundant than in *B. pertussis*. For the former, it may be helpful to be able to switch between profoundly different metabolic states, using BvgAS-dependent gene regulation to rapidly alternate the Bvg^+^ and Bvg^−^ phases. For *B. pertussis*, which has no environmental phase, the purpose of the Bvg^−^ phase is not known. It is possible that the Bvg^−^ phase of *B. pertussis* is an evolutionary remnant, since it shares a common ancestor with *B. bronchiseptica*. Alternatively, the avirulent state of *B. pertussis* may still play a role in the infection cycle, which is yet to be discovered. The first hypothesis is consistent with the observation that virulence factors expressed in the Bvg^+^ phase are sufficient for infection in animal models. However, since the Bvg^−^ phase has been conserved in *B. pertussis*, at least in part, it may be responsible for more than turning off virulence factor production, despite the absence of an environmental phase of growth. Many genes involved in multiple metabolic pathways are negatively regulated by the BvgAS system and are thus upregulated in the Bvg^−^ phase [107]. They include genes involved in lipid metabolism, the TCA cycle, amino acid transport systems, peptidoglycan synthesis and the glycolate oxidation pathway. Thus, *B. pertussis* undergoes multiple metabolic changes when shifting between Bvg phases. It may be that metabolic genes are up-regulated in the Bvg intermediate phase (Bvg^i^) in conditions such as those encountered in the nasal cavity. The resulting metabolic changes might favor maximal bacterial proliferation during the catarrhal phase to optimize the probability of transmission via aerosol droplets. However, the Bvg^i^ phase is poorly understood and more work is required to link specific gene expression changes in this phase to distinct steps in the infection cycle.

*B. pertussis* genes involved in cellular respiration, cell division, cell wall synthesis and sulfur metabolism are expressed at lower levels in mice than *in* *vitro* [108]. This observation suggests that the bacteria grow more slowly *in* *vivo* than *in* *vitro* or/and that the metabolism is altered *in* *vivo*. Although this study highlighted the difference between *in* *vitro* and *in* *vivo* metabolism, many aspects of the *B. pertussis* metabolism during infection, including its carbon source *in* *vivo*, still remain unknown.

In addition to transcriptomic studies, essential gene studies can help elucidate basic physiology of an organism, both *in* *vitro* and *in* *vivo*. Transposon-directed insertion sequencing (TraDIS) is a powerful tool that has been used to identify genes involved in viability in different growth conditions. As an example, genes can be essential depending on whether the bacteria were grown in the Bvg^+^ or Bvg^−^ phase [109]. Interestingly, that study showed some of the genes involved in cell wall synthesis are essential only in the Bvg^−^ phase. This is the case for the entire *mre* locus and genes involved in energy metabolism, including all four genes involved in synthesizing the succinate dehydrogenase complex. Such studies have shown that conditionally essential genes of *B. pertussis* depend on the Bvg phase, and entire processes, such as cell wall biosynthesis, can proceed differently depending on the Bvg phase. These observations provide further evidence that BvgAS not only regulates virulence but is a key regulator in wider growth and metabolism, implying coordination between virulence and metabolism for *B. pertussis*. It should be noted that the Bvg^−^ phase described in [109] was obtained using 50 mM MgSO_4_, which might by itself have some impact on gene expression.

Another technology, termed Tn-seq, has been used to discover *B. pertussis* genes that are essential for growth *in* *vivo* [110], during infection in mice. At day 3 post-infection, more genes were found to be conditionally essential than at day 1 after infection. Interestingly, only few classical virulence factors were identified as essential in these conditions, probably due to redundancy in their function. Of the essential BvgAS-regulated genes, the majority were associated with metabolism, illustrating the importance of a coordination of metabolism and virulence by BvgAS *in* *vivo.* In fact, most genes required for infection are associated with metabolism, regardless of regulation by BvgAS, and include genes involved in transport and biosynthesis of secondary metabolites, many of unknown function. An example of genes which were found to be essential on day 3 but not day 1 post-infection is the *bhu* operon, encoding a heme iron acquisition system, which shows that the process of acquiring iron from heme at this stage in infection is critical [111]. Furthermore, the *cyoABCD* operon encoding a quinol oxidase is also essential only *in* *vivo*, which may be due to adaptation to low oxygen concentrations in these conditions.

Through functional genomic studies it has thus become clear that metabolism is coordinated during infection by *B. pertussis*, at least partly in tandem with virulence through the BvgAS system. These studies have provided valuable insight into an organism which has adapted its metabolism to its host environment, but which has still retained the ability to change its metabolism and adapt to the changing parameters during infection, potentially to optimize growth and enhance transmission.

### Interaction with host metabolism: Hyperinsulinemia and metal acquisition

Some virulence factors of *B. pertussis* may have effects on host metabolism. Very early studies [112] reported hypoglycemia in infants with pertussis. This was confirmed in more recent studies showing that insulin levels are higher in the patients with pertussis than in controls [113]. Mice infected with *B. pertussis* also develop hyperinsulinemia which led to a state of hypoglycemia [114]. PTX is likely to be a key virulence factor responsible for these observations, as it induces insulin secretion by pancreatic β cells and has been referred to as islet-activating protein [115–117]. Injection of PTX into diabetic rats leads to normalization of glucose tolerance [116]. In humans, insulin levels were enhanced following a single injection of PTX, but glucose concentrations were not modified, indicating that hyperinsulinemia in humans is not necessarily accompanied by hypoglycemia. Nevertheless, these studies show a systemic effect of PTX during infection in humans and suggest that this toxin is able to play a role in changing the host metabolism. Hypoglycemia in *B. pertussis*-infected patients may potentially diminish the capacity of their immune response to effectively combat the infection, since activated immune cells require enormous amounts of glucose for their effector functions [118–120]. Effective inflammatory responses often lead to insulin resistance, in order to augment glycemia to fuel their energy requirements. PTX may thus counterbalance this mechanism through its hyper-insulinemic effect.

Another metabolic adaptation of *B. pertussis* to its host is its ability to acquire essential, host-sequestered oligo-elements, such as iron and copper. *B. pertussis* gains iron from host proteins through the production of siderophores [121,122], such as alcaligin, whose synthesis is negatively regulated by the presence of iron. Mutants defective in siderophore synthesis are affected for growth and survival in mouse models of infection [123]. *B. pertussis* also acquires iron from heme [111], which occurs through the Bhu system. The expression of the *bhu* gene is iron-regulated and specifically heme-inducible [124]. Bhu-deficient mutants have a fitness defect in prolonged mouse colonization, providing evidence for a crucial role in iron acquisition from heme especially during later stages of infection [125]. A study on the regulation of siderophore production during infection in mice confirmed that alcaligin is important early in infection and that the Bhu heme system is important later in infection, probably reflecting the changing availability of iron during infection, perhaps as the specific niche in the respiratory tract changes with time [126]. An *in* *vitro* transcriptomic study carried out in iron-starved conditions revealed a previously undescribed ferric iron transport system, FbpABC, which is iron-repressed [127]. Subsequently, a ferrous iron transport system named FtrABCD was identified and shown to be active especially in acidic conditions *in vitro*, suggesting adaptation to acidic microenvironments during infection [128], when siderophores may become ineffective. Thus, the expression of genes which *B. pertussis* uses to gain iron is tightly controlled and appears to change in a temporal fashion during infection.

Transcriptomic and proteomic studies have revealed the interplay between iron metabolism and virulence [127,129]. Upregulation of genes under iron starvation causes greater attachment to epithelial respiratory cells *in vitro*, as a result of increased mucin binding [129]. Furthermore, the genes coding for the T3SS are up-regulated during iron starvation [127]. In contrast, several other virulence factors, including FHA, fimbriae, Prn and ACT are less abundant under iron-starved conditions, which was speculated to decrease the recognition of the bacteria by immune cells in conditions in which the bacteria may be stressed [129]. The gain of iron appears thus to be linked to the production of virulence factors, providing further evidence for the coordination of metabolism and virulence. During iron starvation, *B. pertussis* is metabolically stressed and, in addition to upregulating *bhu* expression and siderophore production, it increases the production of poly-hydroxybutyrate, a storage compound found previously to build up when growing in an excess of carbon source [130].

Like iron, copper is an essential element in most organisms because of its role as a cofactor in various proteins. However, the toxicity of this metal is also used by protozoa and phagocytic cells to poison ingested bacteria [20,131]. Copper homeostasis systems found in bacteria are correlated with their lifestyles [132]. *B. pertussis* harbors genes of copper homeostasis classically found in free living bacteria, but most of them are inactivated by insertion sequences, unlike in *B. bronchiseptica*. The only system active in *B. pertussis* is a copper-induced tolerance system composed of a metallochaperone for copper passivation and two peroxide detoxification enzymes [133]. This system is probably an evolutionary remnant of mechanisms developed to survive amoebal predation that nowadays help the bacteria to survive immune phagocytosis in mammals [134]. Interestingly, *B. pertussis* and other *Bordetella* species possess an original copper-regulated operon for uptake of this metal, suggesting that the bacteria might actually be starved of copper in certain host microenvironments (A.R.-M. and F. J.-D., unpublished data).

## Creation of a privileged niche in naïve hosts

### Interactions with the respiratory epithelium

*B. pertussis* is a strictly mucosal pathogen. It efficiently colonizes the upper respiratory tract of the human host. Infection of the lower respiratory tract can accidentally occur during severe pertussis [135]. In the first steps of infection by *B. pertussis* adhesins, principally FHA and fimbriae, mediate bacterial attachment to the respiratory epithelium. The N-terminal part of FHA contains a heparin-binding domain that interacts with sulfated glycolipids and proteoglycans, key components on the surface of epithelial cells and the extracellular matrix [136]. FHA contains an RGD (arginine-glycine-aspartate) motif, which has initially been proposed to allow *B. pertussis* to attach to integrin CR3 on the surface of monocytes [137]. However, direct binding studies with purified CR3 have shown that, while FHA is able to bind to CR3, this binding is not directly mediated by the RGD sequence [138]. Instead, the RGD sequence of FHA directly interacts with the leukocyte-response integrin and integrin-associated protein (LRI/AIP), which upregulates CR3 binding activity [139]. A carbohydrate-recognition-domain mediates adherence to ciliated epithelial cells [68,140]. Finally, the C-terminal domain beyond the β-helical region is involved in adherence to certain cell types such as rat lung epithelial (L2) cells and J774A.1 macrophages [141].

The minor *B. pertussis* fimbrial subunit FimD has been shown to bind to very late antigen-5 (VLA-5) on the surface of monocytes, and the interaction of FimD with VLA-5 activates CR3 through protein tyrosine kinase signaling, which may enhance FHA-mediated *B. pertussis* binding [142]. Studies in mice have shown that FimD is important for the colonization especially of the trachea and the lungs [143].

The role of Prn in colonization of the respiratory tract is unclear, as there is little convincing evidence of its importance for adherence to epithelial cells [144]. Experiments with *B. bronchiseptica* have shown that the loss of pertactin leads to a defect in colonization only in SCID mice [145], which rather suggests interactions with immune cells. The tracheal colonization factor Tcf is a poorly characterized virulence factor exclusive to *B. pertussis*. It also contains integrin-binding RGD motifs and seems specific for colonization of the trachea [75].

Adhesins are not the only mediators of interactions with epithelial cells. Other virulence factors such as ACT, TCT and the T3SS also indirectly interact with the epithelium to allow the bacteria to persist in its niche. TCT plays a major role in the disruption of the epithelium. By inducing lysis of ciliated epithelial cells and overproduction of mucus, it enables the bacteria to avoid mucociliary clearance [87]. The enzymatic activity of ACT targets regulatory pathways of cells and also disorganizes the epithelium by altering the tight junctions [146]. Moreover, ACT physically associates with FHA, which inhibits biofilm formation *in* *vitro* [147]. The T3SS also induces cytotoxicity in the respiratory epithelium [91,92], although *B. pertussis* BteA is less potent than that of *B. bronchiseptica* [93].

#### Strategies to escape recognition by naive hosts: A question of balance

In addition to specific interactions with the respiratory epithelium, *B. pertussis* has also evolved mechanisms to escape recognition by the first line of immunity, in particular by innate immune cells, without completely silencing the host immune response. To achieve this delicate balance, *B. pertussis* has modified surface molecules to decrease their immunogenicity, such as LPS that composes the outer leaflet of the outer membrane of Gram-negative bacteria. LPS is made of a lipid A membrane anchor, an oligosaccharide core and a polysaccharidic O antigen, and is a strong triggering molecule of the innate inflammatory response. The *B. pertussis* LPS-like molecule, named LOS, is devoid of O antigen, and therefore is less inflammatory and immunogenic than LPS. The presence of an unrepeated tri-saccharide composed of α-N-acetylglucosamine, β-2-acetamido-3-acetamido-2,3-dideoxy-mannuronic acid and β-l-2-acetamido-4-methylamino-fucose protects the bacteria from the bactericidal effect of the pulmonary surfactant proteins SP-A and SP-D [148,149]. In addition, the reactogenicity of the lipid A moiety of *B. pertussis* LOS is further decreased thanks to the inactivation of *pagP*, the gene coding for a transferase that adds a palmitic acid residue to lipid A. The absence of the palmitate reduces LOS recognition by the innate immune Toll-like receptor 4 (TLR4) [150]. Furthermore, in the Bvg^+^ phase the phosphate of lipid A is substituted by glucosamine [151], which intriguingly slightly increases the endotoxic activity of *B. pertussis* LOS and results in the induction of the pro-inflammatory cytokines IL-6, IL-1β and TNF-α [152,153]. However, at the same time, this modification enhances the resistance of the outer membrane to cationic antimicrobial peptides [154].

Together with surface modifications, *B. pertussis* also forms biofilms providing protection against the immune system. The architecture of this biofilm is complex. Major components include FHA, the surface-associated polysaccharide poly-β-1,6-N-acetyl-d-glucosamine produced by enzymes encoded by the *BpsABCD* genes [155] and extracellular DNA [156]. In a mouse model of infection, bacteria were found in biofilms up to 19 days post-inoculation in the nasal cavity, suggesting a role of biofilms for bacterial persistence [156].

## Subversion of anti-*B.* *pertussis* Immunity

### Subversion of innate immune responses

In addition to surface modifications and biofilm formation to decrease its visibility by the immune system, *B. pertussis* has evolved strategies to subvert the innate immune defense mechanisms and re-shape both innate and adaptive immune responses. Resident cells, infiltrating monocytes and immature DCs are the first immune cells to sense *B. pertussis* molecules via recognition by TLR and NOD and NOD-like receptors [157,158]. Activated immune cells and infected epithelial cells release cytokines and chemokines that mediate recruitment and activation of polymorphonuclear neutrophils (PMN), macrophages, natural killer (NK) cells, and certain types of T lymphocytes [159,160]. Activated innate cells ingest and kill bacteria and therefore help to clear the infection, while antigen-presenting cells (APCs) provide a link between innate and adaptive immunity important for long-term protection against (re)infection. The strategies deployed by *B. pertussis* to overcome these lines of defense comprise 1° the inhibition of cell trafficking to the respiratory tract, 2° the suppression of phagocyte activation, 3° triggering cell death and 4° blocking complement-mediated killing.

Among phagocytes, PMNs are the most numerous and have the greatest bactericidal capacity. Despite activation of neutrophil recruitment pathways in mice within 6 hours after *B. pertussis* infection, neutrophil influx in the lungs is delayed up to 3 days [159,161]. This blockage of neutrophil migration is mainly due to PTX, which prevents early neutrophil chemotaxis by inhibiting the production of attracting chemokines released in response to TLR-4 activation of alveolar macrophages (AM) and probably epithelial cells [162]. Decreased chemokine release may also contribute to the reduced early infiltration of monocytes and lymphocytes in the airways [163,164]. Likewise, TCT and ACT may inhibit trafficking of professional phagocytic cells to the respiratory tract [165–167].

Monocytes that arrive at the site of infection differentiate into functional effector macrophages to replenish the resident sentinel cells. However, exposure of primary human monocytes to ACT inhibits their differentiation [168], which makes them poorly phagocytic and devoid of pseudopodia. *In vitro*, ACT also causes de-differentiation of primary human alveolar macrophages into monocyte-like cells with upregulated CD14 levels [168]. Reprogramming of infiltrating monocytes and alveolar macrophages to less bactericidal and short-lived monocyte-like cell types might be particularly relevant to infection of infants and the elderly, whose immune defense relies strongly on innate immunity, with monocytes and macrophages playing significant protective roles.

Several circulating and membrane proteins of innate immune cells collaborate with TLRs in sensing microbial ligands and promoting inflammatory responses [169]. Phagocytic cells that encounter *B. pertussis* recognize the bacterium via surface-exposed molecules or surface-bound opsonins, such as immunoglobulins and complement proteins. Upon phagocytosis, the route of intracellular trafficking determines the fate of internalized *B. pertussis*. In the absence of opsonizing serum, *B. pertussis* might be able to shift the balance toward the non-killing CR3-mediated internalization, thereby escaping clearance by the more efficient Fc receptors (FcR)-mediated pathway [137,170].

Non-opsonic recognition of *B. pertussis* by CR3 depends on the fibronectin receptor VLA-5 that binds to FimD, and the CR3-VLA-5 complex increases CR3 binding via FHA [142,171]. Interaction of FHA via its RGD motif to the LRI/IAP, mediating is cross-linking, also results in upregulation of CR3 and enhances CR3 recognition by an unidentified FHA domain [139,141]. It is likely that FHA adhesion to the cells promote cell intoxication with ACT that notably blocks the oxidative burst capacity of neutrophils [172].

When bacteria are internalized through the non-opsonic route, a significant fraction of macrophage-phagocytosed *B. pertussis* can prevent phagosome-lysosome fusion and eventually replicate in compartments with early endosomal characteristics. This escape mechanism involves microtubule assembly, lipid raft integrity, and the activation of a tyrosine-kinase-mediated signaling [27–29]. Deletion of the *mgtC* gene leads to a higher proportion of bacteria trafficked to lysosomes, suggesting that MgtC may enable the bacterium to prevent phagosome-lysosome fusion [173]. The enzymatic activity of ACT also appears to extend the intracellular survival of non-opsonized bacteria internalized by human and murine macrophages [174,175], while inhibiting opsonophagocytis in primary neutrophils [176]. This indicates that cAMP activates signaling pathways that result in the blockade of phagocytic killing of bacteria [175–177]. ACT was further reported to block phagocytosis by hijacking RhoA activity and by blocking Syk signaling and membrane micropinocytosis [178–180].

PTX inhibits the antimicrobial activity of resident airway macrophages [181] and can also suppress the phagocytic activity of monocytes [182]. It may also intoxicate other resident cells in the lung tissues and inhibit their secretion of neutrophil-attracting chemokines, thereby significantly reducing neutrophil recruitment [162]. Furthermore, the administration of PTX to mice strongly reduced the amplification of the inflammatory response of TLR-stimulated epithelial cells and macrophages by serum or serum-borne lipids, such as lysophosphatidylcholine, confirming an anti-inflammatory activity of PTX [169].

Among key strategies to subvert host immune defenses, *B. pertussis* also induces phagocyte apoptosis via ACT-mediated activation of the macrophage effector caspases 3 and 7 [183,184]. The pro-apoptotic signaling of ACT depends on its enzymatic activity [185,186]. ACT‐produced cAMP also triggers macrophage apoptosis by the mitochondrial route [185]. Programmed cell death is further promoted by the ability of ACT to form cation-selective pores in the cell membranes [187]. *B. pertussis* FHA might also affect apoptosis. Purified FHA was shown to induce dose-dependent apoptosis in human phagocytic and epithelial cells, possibly through the inhibition of the NF-κB pathway [188,189]. However, this effect may be nonspecific as purified FHA is generally contaminated with LOS, a potent TLR-2 ligand that triggers apoptosis via TNF-α secretion.

*B. pertussis* has also evolved defense mechanisms against the complement system. By interacting with the C1 complex, the autotransporter BrkA prevents the deposition of the C3, C4 and C9 proteins on the bacterial surface thereby inhibiting both the classical and the alternative complement pathways [190]. Moreover, *B. pertussis* hijacks two proteins, C4BP and C1INH, that block the complement cascade. C4BP is recruited to the bacteria via FHA [191], and C1INH is subverted by the autotransporter Vag8 [77,192,193]. ACT also plays a role in complement resistance, by preventing complement-mediated opsonophagocytosis. It invades cells carrying CR3 and diverts the phosphatase–dependent dephosphorylation pathway, thereby inhibiting the complement-dependent oxidative burst and inducing non-apoptotic cell death [175,178,194].

Thus, *B. pertussis* deploys an arsenal of effectors that subvert innate immune responses. On the other hand, it has evolved specific adaptations that reduce its capacity to suppress the host inflammatory response via modifications in effector molecules that are transported by the T3SS. In *B. bronchiseptica*, the T3SS BteA effector is necessary and sufficient for cytotoxicity *in* *vitro* [195,196], inducing necrosis through an actin cytoskeleton signaling pathway and inhibiting phagocytosis by macrophages [92]. On the other hand, BteA was reported to interfere with NF-κB to modulate host immune responses [197]. In *B. pertussis* BteA has very low specific cytotoxic activity *in* *vitro* due to the insertion of one Ala residue in its sequence [93], and furthermore the T3SS expression is tightly regulated by the anti-σ factor BtrA. Deletion of *btrA* in *B. pertussis* derepresses the T3SS genes and confers readily detectable BteA-dependent cytotoxicity *in* *vitro* [52]. It is thus likely that the reduced cytotoxic activity of the *B. pertussis* T3SS results from a combination of the inhibition of its expression by BtrA activity and of the reduced specific activity of BteA. In line with this, the deletion of the additional Ala residue increased lethality of *B. pertussis* in a mouse model of intranasal infection and decreased inflammation in the mouse lungs at sublethal doses of infection, most likely due to its immunosuppressive activity [93]. The latter observation suggests that the reduced activity of *B. pertussis* BteA might contribute to the acute course of the pulmonary form of human infant pertussis.

### Subversion of innate control of adaptive immunity

As the infection progresses the bacteria are challenged by the adaptive immune response. The orientation of the adaptive immune response depends on the primary responses induced by epithelial cells and innate immune cells, in particular DCs as powerful APCs. Both humoral and cellular immune responses contribute to protection against pertussis.

After recognition by bacterial TLR ligands immature intra-epithelial DCs mature and migrate to lymphoid tissues, where they present pathogen-derived antigens to naïve CD4^+^ and CD8^+^ T cells. This immunological synapse initiates the adaptive T cell response by priming the expansion and differentiation of naïve CD4^+^ T cells into different T-helper cell subsets, among which the Th1 and Th17 cells are the most important subsets for protection against *B. pertussis* [198,199]. There is strong evidence that Th1 cells are responsible for effective clearance of *B. pertussis* from the lungs of infected mice, while Th17 cells – and in particular resident memory IL-17-producing CD4^+^ T cells – mediate clearance from the nasal cavity [199–202]. They do so by mobilizing neutrophils probably through granulopoeisis and chemotactic cytokine and chemokine induction, as well as by increasing neutrophil survival [203]. IFN-γ produced by Th1 cells mediates activation of neutrophils and macrophages, triggers the formation of opsonizing antibodies and enhances FcR and CR expression on myeloid cells. It also plays a role in the induction of HLA class II expression on the cell surface. Antigen processing and presentation by class II HLA activates CD4^+^ helper-T cells, that promote the maturation and differentiation of antigen-specific B cells and the production of antibodies that are critical for preventing reinfection.

*B. pertussis* is apt at perturbing the deployment of the adaptive immune response by significantly delaying Th17 responses in the blood and in the lungs of mice [204], which is caused in part by the T3SS [205]. Moreover, internalization of *B. pertussis* by myeloid cells triggers the release of IFN-α, which directly suppresses IL-17 responses [204,206]. In addition, FHA-triggered IFN-α responses by peripheral blood mononuclear cells also compromise Th17 cell differentiation and potentially promote suppressive T cells [204]. In parallel, PTX prevents the migration of IFN-α-expressing DCs from the lungs to the lymphoid tissues [207], and thereby enriches the local IFN-α environment able to reduce the Th17 responses, which facilitates bacterial colonization.

At the peak of infection, PTX stimulates pro-inflammatory responses, including Th17 responses in the airways, and these responses are long-lived [208]. In addition to exacerbating airway inflammatory responses at the peak of infection in mice, PTX also inhibits the host mechanisms to resolve inflammation [209]. The local Th17 responses trigger the recruitment of neutrophils, which has been associated with cough, bronchial hyperactivity and hypersecretion in lung diseases [210]. This neutrophil influx may prolong paroxysmal cough typical for *B. pertussis* infection in humans. Eventually, however, Th17 and associated cytokines, such as IL-22, may potentially synergize with IL-17 to control infection [211]. Th17 cells play a crucial role in the resolution of infection and in memory immune responses.

To avoid protective Th1 responses, several *B. pertussis* virulence factors alter the priming of T cells. Through its enzymatic activity ACT perturbs the T-cell activation cascade [212,213] by disassembling the immune synapse [213,214]. In addition, ACT can intoxicate DCs to alter the Th1/Th17 balance and limit Th1 expansion in mice [215–217]. ACT activity also strongly interferes with human monocyte-derived DC functions [218], decreasing their capacity to present antigens to CD4^+^ T cells [219] and to induce CD8^+^ T cell proliferation, while enhancing IL-10 and IL-17-production [220]. Both ACT and PTX promote innate IL-1β production by myeloid cells through the activation of the NALP3 inflammasome and caspase-1 [221,222]. Interestingly, ACT-mediated activation of caspase-1, which polarizes the T cell response toward the Th17 subtype and promotes clearance of the bacteria, did not depend on the enzyme activity of this toxin but was due to its pore-forming activity [221]. In parallel, increased levels of cAMP can enhance the capacity of DC to promote regulatory T cells [220], which may alter protective immune responses against *B. pertussis*.

In addition, intoxication of epithelial cells by ACT during the course of infection impacts their cross-talk with resident immune cells [223]. As mucosal epithelial cells respond to IFN-γ, the presence of IFN-γ in the airways likely influences how they regulate downstream immune responses, including the recruitment of NK cells and T cells [224,225]. ACT exposure enhances IL-6 secretion by broncho-tracheal and nasal epithelial cells [223], a pleiotropic cytokine that promotes Th2 differentiation and simultaneously inhibits Th1 polarization [226]. IL-6 also plays an important role in regulating the balance between Th17 cells and regulatory T cells [223,227].

Other *B. pertussis* virulence factors suppress IL-12 production and skew the response toward anti-inflammatory cytokines. McGuirk and Mills have provided evidence that FHA inhibits the secretion of IL-12 by LPS-stimulated macrophages through upregulation of IL-6 and IL-10, thereby avoiding protective Th1 immunity and favoring the generation of tolerogenic regulatory T cells [228]. However, it has also been reported that this effect might be due to the presence of contaminating LOS [229]. Other studies have shown that wild type FHA can induce the secretion of IL-10 by human monocyte-derived dendritic cells, even in the presence of LOS-neutralizing polymyxin B, while a truncated version of FHA did not, even in the presence of LOS [230]. These studies suggest a role of FHA in the induction of regulatory cytokines by innate immune cells, perhaps in synergy with LOS. Fedele et al. have indeed shown that *B. pertussis* LOS can modulate human dendritic cell function and influences T cell polarization [231,232]. Prn also has an immunomodulatory function and dampens the production of pro-inflammatory cytokines by DCs [233]. Finally, the T3SS effector BopN may facilitate survival of *B. pertussis* by inhibiting local innate inflammatory responses and the subsequent induction of antigen-specific Th1, Th17, and antibody responses [205]. In DCs BopN can also induce the expression of IL-10 through the inhibition of both extracellular signal-regulated kinase [95] and NF-κB pathways.

**Figure 2.**
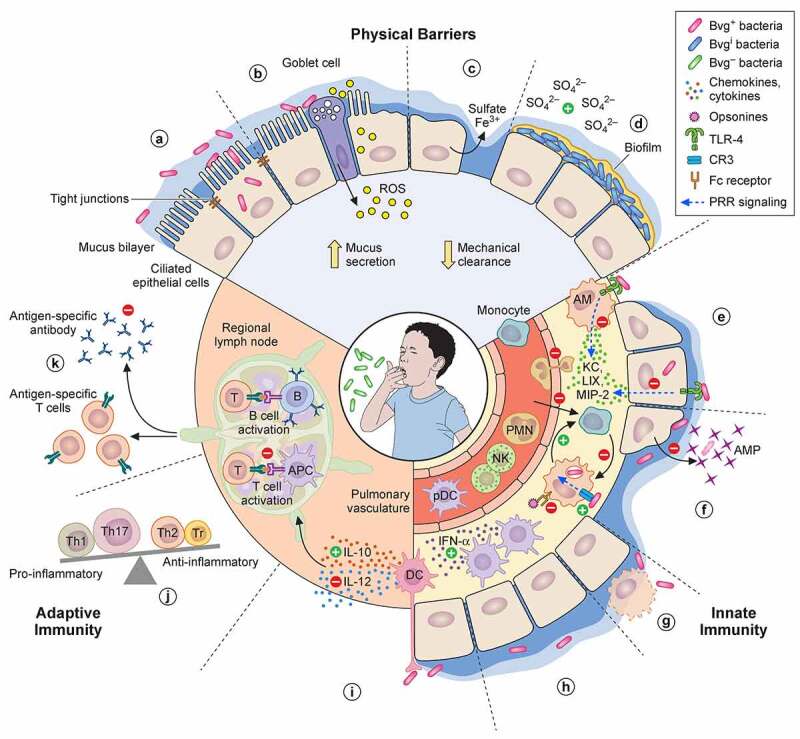
**Anti-immune strategies of *B. pertussis***. (a) Upon infection, several virulence factors (FHA, Prn, Tcf, Fim, BteA, ACT, PTX) regulated by the BvgA/S two-component system (Bvg^+^ bacteria) enable *B. pertussis* adherence to epithelial cells of the nasal mucosa. The role for ACT in adherence is likely indirect. Some bacteria may invade epithelial cells. (b) TCT is internalized by epithelial cells and, along with LOS, induces the release of NO by the intoxicated cells, which is a major destructor of epithelial cells. ACT and BteA may also contribute to the disruption of the epithelial barrier. (c) Once the epithelial barrier is broken and submucosal glands have fired, iron may be delivered onto the surface of epithelial cells by the plasma exudate [234]. In response to the infection, the respiratory epithelium may produce highly sulfated mucins. Sulfate might be released from mucins by glycosyltransferases of *B. pertussis* and commensal bacteria [235,236], which might induce the Bvg^i^ phase and hence trigger biofilm formation (d), thereby allowing long-term colonization. Up-regulation of the pendrin anion exchanger of epithelial cell drives the production of mucus, which along with ciliary damage, reduces mechanical clearance of the bacteria. (e) *B. pertussis* toxins, in particular PTX, suppress the early recruitment of immune cells. PTX prevents neutrophil chemotaxis indirectly by inhibiting the production of neutrophil-attracting chemokines KC, LIX and MIP-2 by alveolar macrophages (AM) and epithelial cells following disruption of TLR-4 signaling. (f) ACT may elicit reprogramming of infiltrating monocytes or alveolar macrophages to less bactericidal and short-lived monocyte-like cell types. Alternatively or additionally, FHA may enhance non-opsonic CR3-phagocytosis to escape effective clearance mediated by FcR recognition and suppresses – along with BrkA, LOS and ACT, and most of all Vag8 – complement-mediated killing (not represented). Expression of MgtC may enable the bacterium to escape opsonophagocytic killing by preventing phagosome-lysosome fusion, while ACT, PTX, FHA and Prn dampen the antimicrobial activity of the phagocytes, including oxidative stress and release of antimicrobial peptides (AMP). (g) To further avoid clearance by professional phagocytes, *B. pertussis* virulence factors such as ACT could trigger phagocyte apoptosis. (h) Several virulence factors may contribute to the accumulation of IFN-α-expressing DCs in the lungs that mediate early suppression of Th17 cell differentiation. (i) Intraepithelial DCs (and other myeloid cells) are reprogrammed to up-regulate IL-10 and down-regulate IL-12 production in order to favor the differentiation of regulatory T cells (Tr), Th2 and Th17 cells over Th1 cells. (j) ACT-intoxicated DCs are impaired in their capacity to stimulate T cells. The arrival of potentially tolerogenic DCs into the draining lymph nodes may alter cellular protective immune responses against *B. pertussis*. (k) Additionally, PTX is able to suppress serum antibody responses

Overall, the *B. pertussis* anti-immune strategies summarized in Figure 2 allow the bacteria to very efficiently colonize the respiratory tract of susceptible hosts, which may explain its high contagiousness. It is possible that factors other than those described here may also modulate adaptive immune responses to *B. pertussis*, but they require further investigation. There is also a significant gap in our knowledge of how preexisting immunity may affect immune responses upon re-infection. An effort in identifying adaptive signatures of *B. pertussis* in vaccinated populations is a first step toward understanding escape from preexisting *B. pertussis*-specific immune responses.

### B. pertussis *adaptations to escape preexisting immunity*

Pertussis is rare among recently vaccinated children, but its incidence quickly increases with age [237]. Protection following immunization with aPV wanes faster than that afforded by whole cell pertussis vaccines [238]. aPV used in most industrialized countries are composed of one to 5 different antigens, all combined with tetanus and diphtheria toxoids. All aPV contain detoxified PTX, most contain FHA and Prn in addition, and some further contain serotype 2 and serotype 3 fimbriae. Vaccines with one or more antigens, in addition to PTX, are more effective than vaccines containing PTX only [239]. However, similar to other bacteria for which acellular vaccines are widely used, *B. pertussis* evolves to escape vaccine-induced immunity by modifying the structure or production levels of the antigens present in the aPV.

#### PTX variations

PTX increases the severity of *B. pertussis* infections because closely related *B. parapertussis*, which does not produce PTX, generally causes less severe infections [240]. PTX causes leukocytosis in humans, which correlates with increased rates of hospitalizations and deaths [241]. Since the introduction of aPV, several variations in the *ptx* gene and its promoter region have been identified, some of which have been shown to increase *ptx* expression. In vaccinated hosts, increased PTX production might be beneficial for the pathogen because higher levels of antibodies are required for its neutralization. Seventeen *ptxP* alleles, with mutations in the *ptx* promoter, have been reported in the *B. pertussis* population worldwide [242,243], suggesting positive diversifying selection [244]. In contrast, there is minimal sequence diversity in the *ptx* coding sequences [244–248]. Only five protein variants of the enzymatic subunit PtxA have been identified, of which PtxA1 and PtxA2 predominate [242]. Very few Ptx-deficient isolates have been reported [249–251] so far, and the disease caused by these variants is much less severe than disease caused by PTX-producing strains, confirming the important role of PTX in pertussis pathogenesis [250].

With the progressive disappearance of the pre-vaccination *ptxP2* variant, the *ptxP1* and *ptxP3* variants predominate globally [244,252]. Variants with *ptxP3* promoters have been described to produce increased levels of PTX and were found to be associated with the resurgence of pertussis in the Netherlands [244,252]. They spread quickly and caused enhanced disease in vaccinated and unvaccinated populations. However, the spread and persistence of *ptxP3* variants in vaccinated populations may not necessarily be due to the escape of anti-PTX antibodies but may also be the result of PTX-mediated immune suppression described above.

Compared to the pre-vaccination variants, *ptxP3* strains also differentially express other virulence genes. The expression of the *prn* and *tcfA* genes is enhanced in the predominant *ptxP3* strains in Australia, which may result in improved adhesion, while the downregulation of the T3SS and other immunogenic proteins, such as Vag8 and BipA may reduce immune recognition, which overall may contribute to the increased fitness of these strains [253]. Additionally, mutations in *bscI* – a T3SS gene – found in those strains may be advantageous in reducing immune recognition [253]. It is therefore not possible to ascribe the success of *ptxP3* strains in vaccinated populations solely to enhanced production of PTX due to vaccine pressure.

#### Antigenic variations of Prn, FHA and fim

In contrast to PTX, a substantial sequence diversification has been detected for Prn and FHA, suggesting that *B. pertussis* benefits from changing these proteins in response to vaccine pressure [254]. Thirteen Prn variants have been identified, of which Prn1-Prn3 predominate in *B. pertussis* populations. Prn represents the only antigen of aPV that inducse bactericidal antibodies [255,256], while natural infection with *B. pertussis* induces bactericidal antibodies against a broad range of antigens [256]. Most variations in Prn occur in two regions comprised of five- and three-amino acid repeats [252]. Expansion or contraction of the tandem repeats may affect the binding avidity of anti-Prn antibodies [257].

Strains completely lacking Prn are currently isolated twice as frequently from vaccinated children than from unvaccinated children, and these strains outcompete Prn-producing strains in vaccinated, but not in unvaccinated mice [4,258,259]. The expansion of Prn^−^ strains has reached a prevalence of 78–85% in some regions [260–262]. These findings suggest that the loss of Prn is favorable to *B. pertussis* when a vaccine-induced anti-Prn immune response is present, and that these strains have emerged and expanded because of aPV pressure.

FHA also exhibits diversification in highly antigenic sites but conservation within its functional domains. If diversified sites allow for anti-FHA antibody escape without impacting cytoadherence, this might enhance *B. pertussis* fitness. However, FHA variants have not exhibited the same widespread level of diversity in protein size and presence as Prn [254], most likely because of its importance in pertussis pathogenesis and the fact that current aPV do not induce bactericidal antibodies against FHA. Nevertheless, specific selection of *fhaB* frameshift mutations was found to be most prominent in the lungs of aPV-immunized mice, while they were rarely detected in the noses, suggesting that FHA is more important for colonization of the nose than of the lungs [263].

Like anti-PTX and anti-Prn antibodies, anti-fimbriae antibodies positively correlate with clinical protection [264]. Two variants of *fim2* and 3 variants of *fim3* have been found in circulating strains [252]. The relation between fimbrial types and pathogenicity remains elusive [245,265]. Genomic rearrangements leading to expression of either *fim2* or *fim3* have been observed in some *ptxP3* strains [266], but differences in immune recognition remain untested.

Mutations in *ptxP, prn* and *fim3* are associated with clonal sweep, i.e., these mutations are becoming dominant in the *B. pertussis* population through positive selection, suggesting that they increase strain fitness or are associated with additional mutations that do so [252]. Strain variation was shown to affect vaccine efficacy in mouse models [267–269]. The antigenic divergence observed between vaccine strains and circulating strains may act synergistically with the *ptxP3* polymorphism by enhancing transmission from vaccinated hosts. The recent emergence of strains lacking Prn that also contain the *ptxP3* allele is alarming. However, infected patients who had been vaccinated still experienced less severe forms of the disease than those who had not been vaccinated [248]. Nevertheless, adaptive changes of *B. pertussis* to aPV pressure may increase waning immunity. Of potential concern, an increase in fitness of vaccine-escape strains may lead to a rapid rise in the proportion of cases due to these strains and an overall increase in pertussis case numbers over a 20-year period [270].

## Conclusion: Ongoing evolution of bacterial-host adaptation

The evolution of *B. pertussis* from the *B. bronchiseptica* complex was primarily associated with genome decay, mainly due to recombination between IS element repeat regions. Considering the scale of the gene loss in *B. pertussis*, which results in severe fitness cost in the environment, it is likely that it is a result of sophisticated adaptation to its specific niche, which is the human upper respiratory tract [271]. This high level of adaptation is reflected in the relatively monomorphic nature of *B. pertussis* strains, although adaptation and evolution continue to take place. Thus, recent isolates have undergone further loss of genetic material through insertion elements, the frequency of which is increasing with time [272]. Comparative genomic studies provide evidence of *B. pertussis* changes since the introduction of vaccine programs, in addition to those mentioned above. A study on 343 strains from 19 countries found two distinct branches, although one of them represented only 1.7% of strains, none of which was isolated in recent years [247]. Among the 12 alleles of the *prn* gene, the most diverse virulence factor gene described, three predominate in this collection, and among the eight *ptxA* alleles three are dominant, with *ptxA1* representing 78% of strains tested. However, the *ptxP3* allele frequency has increased overtime, suggesting an increasing advantage of this allele [247].

As described above, the other most striking example of recent continued adaptation by *B. pertussis* to its host is the emergence of strains lacking *prn* as a result of vaccine pressure. The prevalence of these strains varies from 27% of recent isolates from Japan [273] to more than 85% of strains recently isolated in the US [262], and *prn* loss was caused by 10 different types of mutations, including nucleotide substitutions resulting in a stop codon, or insertions resulting in frameshifts. However, the most common mutation was the insertion of IS*481* in approximately 90% of *prn* mutations.

Thus, it appears that *B. pertussis* continues to adapt to its niche, and that the process of restriction to the human respiratory tract by genome reduction is still ongoing. Not unexpectedly, *B. pertussis* continues to adapt to changing human behavior, such as the shift from whole-cell vaccines to aPV, containing only a few antigens. Intuitively, one might expect that this continued adaptation would lead to decreased virulence, especially because of the loss of some of the classical virulence factors. However, this may be counterbalanced by compensating adaptations, such as the emergence of the *ptxP3* allele. Most of such compensating adaptations are still to be discovered.

That the 2012 pertussis outbreak in the United Kingdom was caused by a number of closely related strains rather than the emergence of one hypervirulent clone, argues against specific large-scale genetic changes being responsible for the resurgence in pertussis [274]. In the isolates collected during this outbreak genes encoding antigens included in the aPV evolved faster than other genes encoding cell surface proteins. Although this was already seen before the introduction of the aPV, the rate of evolution of genes encoding aPV antigens has increased since the introduction of aPV.

Evolution of *B. pertussis* is not limited to genome decay but occurs also via genome rearrangement. A recent comparison of the genomes of 469 US strains showed 107 unique chromosome structures [275], with little diversity at the sequence level, indicating that structural rearrangement is also an important source of variability between strains. Chromosomal rearrangements can affect gene expression and may influence the phenotype, as shown in a recent study [276]. Adaptation of *B. pertussis* by genome rearrangement is only just beginning to be understood and its true significance will have to be evaluated through *in vitro* and *in vivo* experiments.

While evolution has enabled *B. pertussis* to adapt very specifically to its host, the host also undergoes evolutionary pressures to resist infection. This evolutionary principle is summarized in the ‘Red Queen” hypothesis [277,278] referring to the work of Lewis Carrol “Through the Looking-glass” where the two protagonists always remain at an equal distance during their chase. In an analogy for coevolution, this theory stipulates that a specific organism in a specific environment must constantly evolve in order to survive, because other living organisms also evolve in the same environment and therefore exert selective pressure on the first one. This hypothesis was extended to the relationship between parasites, more broadly including pathogens and symbionts, and their hosts [279].

Although no study has specifically examined coevolution of humans and *B. pertussis*, some observations prompt us to speculate on the adaptation of the human host to *B. pertussis* infection. Host cells use TLR2 and TLR4 to detect bacterial LPS, but the *B. pertussis* LOS triggers this signaling pathway only poorly [280]. This may be counterbalanced by PTX, which causes activation of TLR2/4 [281]. Likewise, the addition of a glucosamine residue onto the phosphate group of lipid A allows resistance to antimicrobial peptides [151,154], but it also causes increase in IL-6, IL-1β and TNF-α production [152,153]. Furthermore, in synergy with TCT *B. pertussis* LOS triggers the induction of IL-1α and inducible NO synthase (iNOS) in hamster tracheal organ cultures, while either molecule alone does not [282]. TCT is directly recognized by cytosolic peptidoglycan-recognition proteins from insects to mammals, including NOD1, and triggers NF-κB signaling pathways. However, while in NOD1-transfected HEK293 cells, TCT was well recognized by mouse NOD1, it was poorly recognized by human NOD1 [283]. More recent studies have shown that human NOD1 is a sensor for TCT, but TCT must be transported to the cytosol by the peptidoglycan-specific transporter SLC46A2 to activate NOD1 [284], which suggests that humans have adapted their TCT-mediated signaling through a specific mechanism.

The hemolysin RTX domain of ACT forms cation-selective pores by oligomerisation, resulting in efflux of potassium ions from target cells. Perturbation of potassium homeostasis activates dendritic cells and triggers a cell-mediated immune response in the host [177,285,286], thereby turning this property of ACT to humans’ advantage [221].

## Data Availability

I do not have a DOI yet, and since this is an invited signature review, I don’t think this applies here
